# Genetic diversity analysis of some Egyptian *Origanum* and *Thymus* species using AFLP markers

**DOI:** 10.1186/s43141-019-0012-5

**Published:** 2019-12-09

**Authors:** El-Shaimaa Saad El-Demerdash, Esraa Attia Elsherbeny, Yasser Abdelhakim Mohamed Salama, Mohamed Zoelfakar Ahmed

**Affiliations:** Genetic Resources Department, Desert Research Center (DRC), 1 Mathaf El-Matarya Street, El-Matarya B.O.P 11753 Elmatarya, Cairo, Egypt

**Keywords:** AFLP markers, *Origanum* species, Polymorphism information content (PIC), *Thymus* species

## Abstract

**Background:**

In the present investigation, two genera of family Labiatae (genus *Origanum* and genus *Thymus* including their available species in Egypt) were analyzed genetically on DNA level using amplified fragment length polymorphism (AFLP) markers. Four selective primer combinations **(***E-AGC/M-CAA*, *E-AGC/M-CAC*, *E-AGG/M-CTC*, and *E-ACC/M-CAT***)** were used to detect genetic variations (polymorphisms) within and between the studied plant species of each genus and with their cultivated relatives.

**Results:**

A total of 193 amplified fragments were obtained overall primer combinations with a disparity in the number of bands for each primer combination. The primer combination E-AGG/M-CTC exhibited a large number of amplicons (67) compared to the other primers with polymorphism percentage 69%. The polymorphism information content (PIC) was calculated for the four primer combinations showing a very high values ranging between 0.98 and 0.99. On the other hand, *Thymus* species *(Thymus vulgaris*, *Thymus capitatus* L*.*, and *Thymus decassatus* Benth*.*) exhibited a total number of 171 amplicons for all primer combinations with an average of 42 bands. The primer combination E-AGG/M-CTC produced the largest number of bands (62 bands) with polymorphism percentage 35%, even though the primer combination AGC/M-CAA was more efficient to give high polymorphisms within *Thymus* species where it resulted in a total of 35 bands with polymorphism percentage 63%. The PIC values were calculated ranging from 0.96 to 0.99.

**Conclusion:**

One can conclude that AFLP technique was informative and efficient technique to give a good coverage of *Origanum* and *Thymus* genomes. Furthermore, it was helpful to elucidate the genetic variations and phylogenetic relationships within the studied species as a basis for further studies on these genera and related species.

## Background

Family Labiatae or Lamiaceae is a family of flowering plants commonly known as the mint or deadnettle or sage family. It contains about 236 genera and has been stated to contain 7.587 species according to the world checklist of selected plant families (WCSP, 2017). Labiatae plants are frequently aromatic in all parts and include many widely used culinary herbs [34]. In addition, many biologically active essential oils have been isolated from various members of this family. Two genera of family Labiatae were chosen for the present study (*Origanum* and *Thymus*).

Genus *Origanum* comprises about 38 species widely distributed in the Mediterranean, Euro-Siberian, and Irano-Siberian regions [24]. It is an important genus belonging to the Lamiaceae family [10]. This genus is very appreciated for its volatile oil and it is characterized by a great morphological and chemical diversity [15]. *Origanum* species have many potentialities due to their medicinal, culinary, and agricultural importance and will be mentioned briefly hereafter. The medicinal importance includes their effect on gastrointestinal diseases such as diarrhea, stomach pain, colic, and gastric ulcers; respiratory diseases (e.g., asthma, cough and chest pain) [29]; abnormal menstrual cycles [10]; kidney and liver diseases; metabolic, hormonal, and neuronal disorders; skin; and urogenital system diseases [29]. They have also been used as sedatives and, due to their biocidal properties, as antiparasitic and anthelmintic [10, 15]. Furthermore, *Origanum* has been used to control diabetes and obesity such as *O. vulgare* which can delay the development of diabetic complications and correct metabolic abnormalities due to its hypoglycemic property [26]. Moreover, these plants have antibacterial and antifungal activities which give them a very important role not only for treating infectious diseases, but also for use as food preservatives as they much delay microbial growth and better preserved food. Similarly, these plants affect plant and tree fungal pathogens which cause heavy losses to farmers, so they are important for the agricultural sector. On the other hand, although tumors are being very difficult to treat because of its enormous complexity and variability, some *Origanum* species demonstrated antitumor and cytotoxic activity against several cell lines such as (*O. vulgare*, *O. syriacum*, *O. dictamnus*, *O. mycrophyllum*, *O. libanoticum*, *O. majorana*, *O. compactum*, *and O. onites)* as proved by [5–7, 14, 16, 22, 31].

In the same context, genus *Thymus* is very frequent in the Mediterranean region; it consists of over 400 species of herbaceous annual and perennial plants that are extensively used for medicinal and non-medicinal purposes [28]. It has long history of being used in traditional medicine for treatment of various diseases, for instance, to treat respiratory diseases (whooping cough, bronchitis, and asthma) in the form of tea, ointment, tincture, and syrup or by steam inhalation. In addition, it is used to prevent hardening of the arteries, treatment of toothache, urinary tract infection, and dyspepsia [20]. Furthermore, the remedial potential of *Thymus* is due to the presence of flavonoids, thymol, carvacrol, eugenol, phenols, luteolin, and tetramethoxylated. It has several activities such as carminative, antiviral, antispasmodic, antimycotic, mammalian age-delaying properties, antiseptic, antioxidant, anthelmintic properties, anti-HIV-1 activity, antiulcer, hypoglycemic, antihyperlipidemic activity, and specific cytotoxicity against a variety of tumor cell lines [30]. In addition, it acts as a good source of iron, calcium, manganese, and vitamin K and likewise increases blood flow and is used to overcome physical and mental weakness and insomnia [1, 33].

In spite of the wide distribution of *Thymus* and *Origanum* species in Egypt, there are some species that are threatened with loss and extinction such as *Origanum syriacum* which is a very rare plant grown on stony grounds in Sinai Peninsula including the coastal Mediterranean strip [8], and efforts have been undertaken to increase their numbers by cultivation in their natural habitats (Saint Catherine) to protect them from extinction as reported in *Egypt’s Fifth National Report to the Convention on Biological Diversity, 2014*. Additionally, *Thymus decussatus* was listed as a one of the indigenous species threatened with extinction in natural Egyptian environments according to *The State of the World’s Forest Genetic Resources Country Report, Egypt, 2012.* Assi [4] mentioned that *Origanum syriacum* and *Thymus decussatus* are the most collected species for trade in Saint Catherine protectorate because of their medicinal value.

The genetic analysis of plants is considered as a basis for researchers to characterize plant materials in nature, to detect genetic diversity or the genetic homogeneity and to select plants with special characteristics such as production of desired compounds and stress tolerance mechanisms. The pool of genetic variation in plants, namely, the medicinal and the aromatic ones, serves as the base for plant breeding as well as for selection. Molecular markers are very useful in breeding program allowing germplasm screening independent to the developmental stage of the plants and/or environmental factors [27].

Amplified fragment length polymorphism (AFLP) is a powerful DNA fingerprinting technology applicable to any organism of any origin or complexity such as prokaryotes, plants, animals, and human [40]. It was originally described by [41] that the technique combines the reliability of restriction enzyme digestion with the utility of the polymerase chain reaction (PCR). It is based on the selective amplification of genomic restriction fragments by PCR [11].

It is successfully used in several studies for fingerprinting, studying genetic diversity, phylogenetic relationships, and genetic stability in many plant species such as *Murraya koenigii* growing in Eastern Asia Ghosh and Mandi [18], *Kelussia odorotissima* medicinal plant native to the Zagros Mountains in Iran [13], and different varieties of *Curcubita ficifolia* fruit in Mexico [32]. AFLP is a highly sensitive technique to detect genetic variations (polymorphisms) within a species or among closely related species. Population geneticists also use AFLP approaches to determine genetic variation across different populations [19, 44]. Indeed, this method has allowed researchers to refine the taxonomic classification of organisms based on AFLP-associated genetic markers [9]. In addition, AFLP offers several advantages over other currently used DNA markers, such as simple sequence repeats (SSR) and single nucleotide polymorphism (SNPs) [2, 23, 42].

Therefore, the current investigation was carried out to study the genetic diversity among *Thymus* and *Origanum* plant species and to detect genetic polymorphisms and constructing phylogenetic relationships within the studied plant species which could be helpful for an efficient management of these genera.

## Methods

### Plant material and study area

All plant samples were identified and authenticated by Dr. Yousri Abd-Elhady, Ecology and Range Management Department, Desert Research Center (no voucher specimen of this material has been deposited in a publicly available herbarium). The fresh young leaves were collected in spring of 2016, transferred into liquid nitrogen, and kept frozen at − 80 °C till use. The plant species under study are shown later in Fig. 5.

For two *Origanum* species, the cultivated type (*Origanum vulgare*) was collected from private farms at Kirdasa region, Giza Governorate, Egypt, and the other is the wild-type *Origanum syriacum* (L.) *subsp. sinaicum* (Boiss.) which collected from different sites around Saint Catherine Monastery, South Sinai Governorate, Egypt.

The three *Thymus* species chosen for the present study were *Thymus vulgaris* (the cultivated type) which was collected from private farms at Kirdasa region, Giza Governorate, Egypt. For two wild types of thyme, *Thymus capitatus* (L.) was collected from the naturally grown rocky ridge habitats, especially wet sites distributed in the North Coast, Mersa Matruh Governorate, and the other wild type is *Thymus decassatus* (Benth.) which was collected from Saint Catherine Protectorate, South Sinai Governorate.

### Amplified fragment length polymorphisms (AFLP)

The plant samples (young leaves) were ground using liquid nitrogen, and total genomic DNA was isolated using a DNeasy Plant Mini Kit (Qiagen, Santa Clarita, CA). The AFLP analysis was carried out using the AFLP® Analysis System II (Invitrogen, USA) (Cat. No. 10483-022), according to the manufacturer’s protocol. A 400-ng DNA of each sample was digested simultaneously with restriction enzymes (EcoRI and MseI) overnight at 37 °C, the digested samples were incubated at 70 °C for 15 min to inactivate the restriction endonucleases. EcoRI and MseI adapters were ligated to the digested DNA samples to generate template DNA for amplification. Pre-amplification was carried out with EcoRI and MseI primers each carrying one selective nucleotide at the 3′ position. Selective amplification of restriction fragments was carried out using four primer combinations (E-AGC/M-CAA, E-AGC/M-CAC, E-AGG/M-CTC, and E-ACC/M-CAT) (Table 1).

**Table 1:** A list of AFLP primers and adapters sequence

Name	Sequence (5′-3′)
*MseI* forward adapter	GACGATGAGTCCTGAG
*MseI* reverse adapter	TACTCAGGACTCAT
*EcoRI* forward adapter	CTCGTAGACTGCGTACC
*EcoRI* reverse adapter	AATTGGTACGCAGTCTAC
*MseI* primer core region (M)	GATGAGTCCTGAGTAA
*MseI* pre-selective primer (M+1)	M+C
*MseI* selective primers (M+3)	
*M*+CAA	GATGAGTCCTGAGTAACAA
*M*+CAC	GATGAGTCCTGAGTAACAC
*M*+CAG	GATGAGTCCTGAGTAACAG
*M*+ CAT	GATGAGTCCTGAGTAACAT
*M*+CTA	GATGAGTCCTGAGTAACTA
*M*+CTC	GATGAGTCCTGAGTAACTC
*M*+CTG	GATGAGTCCTGAGTAACTG
*M*+CTT	GATGAGTCCTGAGTAACTT
*Eco*RI primer core region (E)	GACTGCGTACCAATTC
*Eco*RI pre-selective primer (E+1)	E+A
*Eco*RI selective primers (E+3)	
*E*+AAC	GACTGCGTACCAATTCAAC
*E*+ACA	GACTGCGTACCAATTCACA
*E*+ACC	GACTGCGTACCAATTCACC
*E*+ACG	GACTGCGTACCAATTCACG
*E*+ACT	GACTGCGTACCAATTCACT
*E*+AGC	GACTGCGTACCAATTCAGC
*E*+AGG	GACTGCGTACCAATTCAGG

### Data analysis

Calculation of the polymorphism information content value (PIC) was based on the results obtained from AFLP according to [38] using the following formula:

$$ \mathrm{PIC}=1-\varSigma {\mathrm{fi}}^2 $$

where fi^2^ is the frequency of the allele. The PIC value shows the discriminatory power of a locus, and it ranges from 0 (monomorphic) to 1 (very highly discriminative).

## Results

**Fig. 1:**
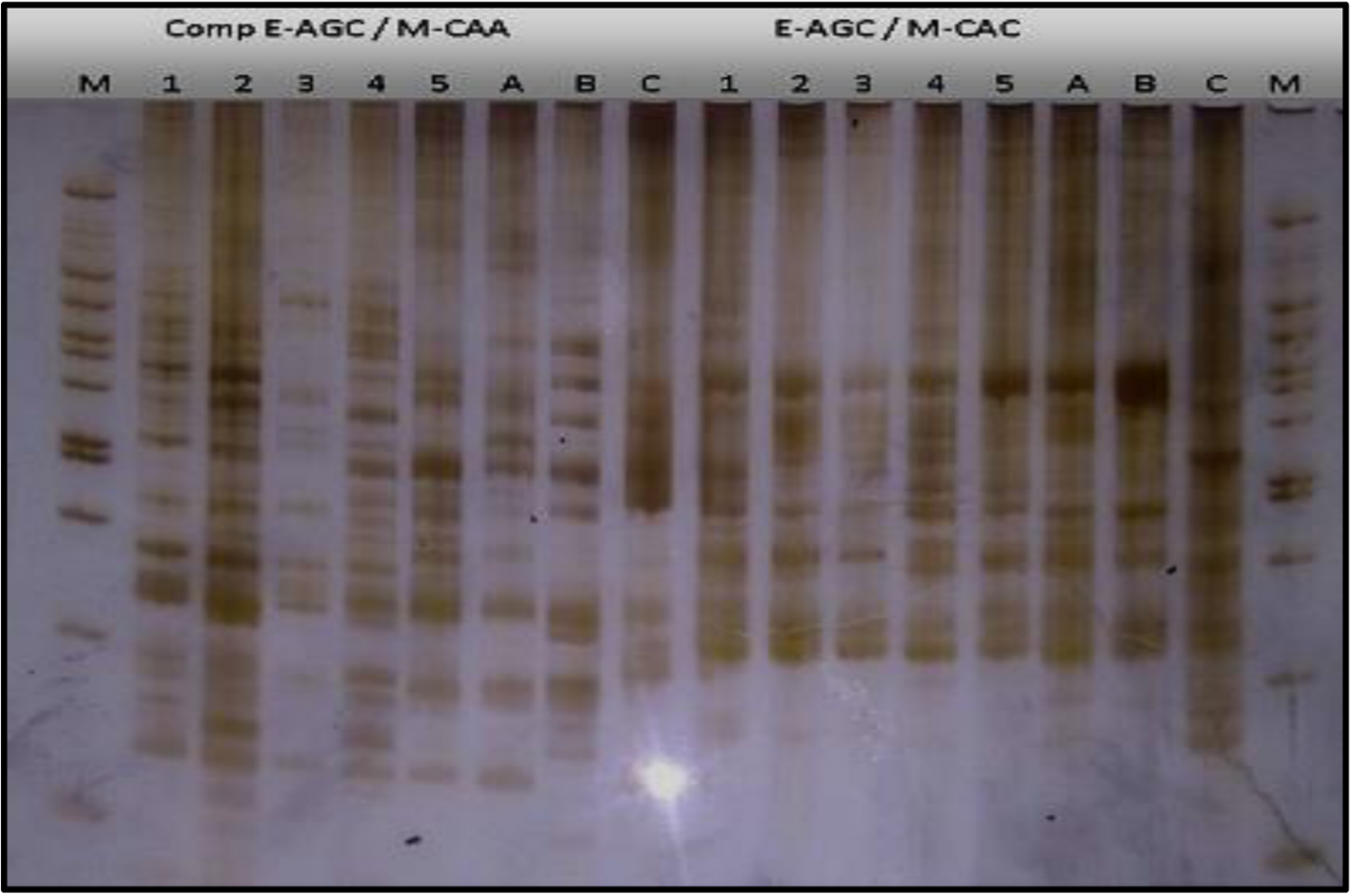
The AFLP banding patterns of *Origanum* species (1, *O. vulgare*; 2, 3, 4, and 5, *O. syriacum*) and *Thymus* species (A, *T. vulgaris*; B, *T. capitatus*; and C, *T. decassatus*) using two primer combinations (E-AGC/M-CAA and E-AGC/M-CAC). M: 100-bp DNA ladder

**Fig. 2:**
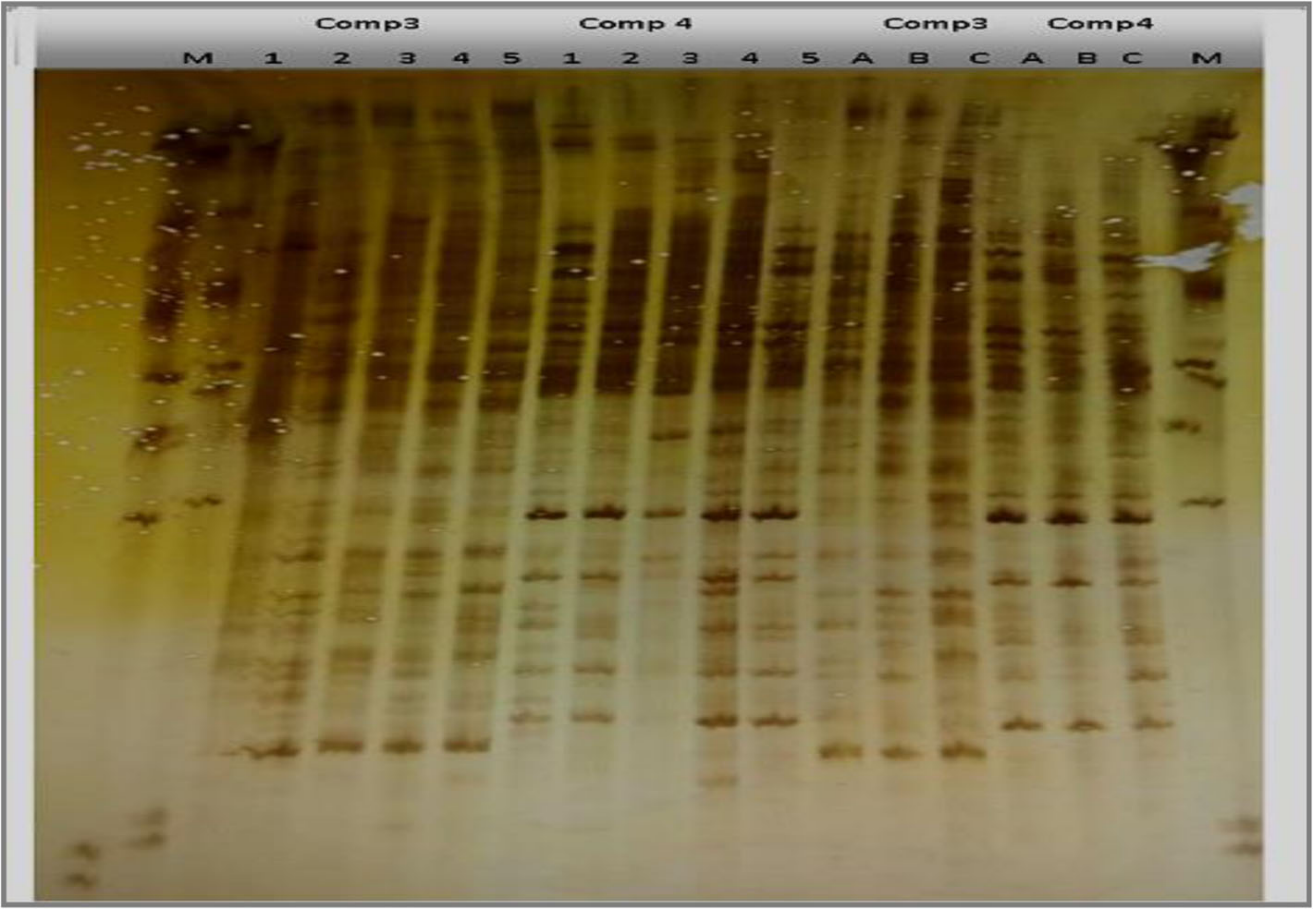
The AFLP banding patterns of *Origanum* species (1, *O. vulgare*; 2, 3, 4, and 5, *O. syriacum*) and *Thymus* species (A, *T. vulgaris*; B, *T. capitatus*; and C, *T. decassatus*) using two primer combinations (E-AGG/M-CTC and E-ACC/M-CAT). M: 100-bp DNA ladder

Four primer combinations were used for studying the chosen *Origanum* and *Thymus* species as shown in Figs. 1 and 2. The resulting data of each species are not scored as length polymorphisms, but instead as presence − absence polymorphisms, and it will be discussed hereafter.

1. *Origanum* species

AFLP profiles showed a large number of bands (193) differing in size and ranging between 105 and 1100 bp with polymorphism percentage 53% for all primer combinations (Table 2). Each primer combination will be presented separately.

- Combination E-AGC/M-CAA

**Table 2:** AFLP primer combinations, selective nucleotides, number of polymorphic bands, total bands, polymorphism percentage, and fragment size range in *Origanum* species

Primer comb.	Selective nucleotides		Number of bands			Polymorphism percentage	Fragment size Range scored
	EcoRI	MseI	Total	Polymorphic	Monomorphic		
E-AGC/M-CAA	AGC	CAA	40	26	14	65%	130–990 bp
E-AGC/M-CAC	AGC	CAC	29	–	29	0%	260–1000 bp
E-AGG/M-CTC	AGG	CTC	67	46	21	69%	115–1100 bp
E-ACC/M-CAT	ACC	CAT	57	31	26	54%	105–1100 bp
Total			193	103	90		
Average			48	25	22	47%	

It produced a total number of 40 bands ranging in size from 130 to 990 bp of which, 14 bands were monomorphic while 26 bands were polymorphic with polymorphism percentage (65%) (Fig. 1 and Table 2). Five positive specific bands (present bands) were detected, out of which two bands at 540 and 200 bp are specific for sample no. 2. Two positive bands at 590 and 380 bp are specific for sample no.5 while the fifth band is at 410 bp, and it is unique for sample no. 4. In contrast, 510- and 390-bp bands were negatively specific (absent bands) for sample no. 5 and likewise for sample no. 2 at 215 bp.

b. Combination E-AGC/M-CAC

This combination resulted in a total of 29 bands ranging between 260 and 1000 bp (Fig. 1). All bands were common (monomorphic) with polymorphism percentage 0% (Table 2). It produced the smallest number of bands relative to the other combinations used.

c. Combination E-AGG/M-CTC

The combination E-AGG/M-CTC produced the largest number of bands (67 bands) compared to the other primer combinations ranging between 115 and 1100 bp with polymorphism percentage 69% (Fig. 2). The monomorphic bands were 21 while the polymorphic ones were 46 (Table 2). Five bands were positive specific, three of which at 250, 200, and 195 bp were unique for sample no. 4 while bands at 210 and 145 bp were unique for samples no. 2 and 3, respectively. On the other hand, eleven negative specific bands were counted, of which bands at 1100 and 170 bp were negatively specific for sample no. 4. Moreover, bands at 900, 680, 600, and 370 bp were absent for sample no. 2 while bands at 480, 310, and 160 bp were negatively specific for sample no. 1 (the cultivated type *Origanum vulgare*) and bands at 180 and 150 bp were absent for sample no. 5.

d. Combination E-ACC/M-CAT

It gave a total number of 57 bands ranging in size from 105 to 1100 bp (Fig. 2). Thirty-one of them were polymorphic bands with polymorphism percentage 54% (Table 2). Eight bands were positively specific for particular samples, of which bands of sizes 1050, 260, 205, and 200 bp were specific for sample no. 1 representing *Origanum vulgare* (cultivated type). Moreover, band at size 1060 bp was specific for sample no. 2 while bands of size 245, 215, and 175 bp were specific for sample no.4. In contrast, only one band at 1000 bp was negatively specific for sample no. 1.

The PIC values were calculated according to the equation mentioned above. The obtained PIC values for *Origanum* species are very high, ranging from 0.98 to 0.99 (Table 3) corresponding to primer combinations E-AGG/M-CTC and E-AGC/M-CAA, respectively with an average of 0.97 all over the four primer combinations. The PIC value provides an estimate of the discriminatory power of a locus by taking into consideration, not only the number of alleles but also the relative frequencies of those alleles. PIC values vary from 0 (monomorphic) to 1 (very highly discriminative, with many alleles in equal frequencies).

2. *Thymus* species

**Table 3:** Information obtained from AFLP using four primer combinations in *Origanum* species

Parameters and their abbreviations		AFLP
Number of primer combinations	*U*	4
Number of non-polymorphic bands	*n*_*np*_	90
Number of polymorphic bands	*n*_*p*_	103
Average number of polymorphic bands/assay unit	*n*_*p*_*/U*	26
Number of loci	*L*	193
Number of loci/assay unit	*n*_*u*_	48
Total number of effective alleles	*N*_*e*_	*221*
Total banding pattern	*Bp*	51
Effective number of patterns/ assay unit	*P*	13
Min range of PIC value	*PIC*	0.9776
Max range of PIC value	*PIC*	0.9863
Average of PIC	*PIC*	*0.9819*
Fraction of polymorphic loci	*β*	0.5336
Assay efficiency index	*A*_*i*_	55.3607
Effective multiplex ratio	*E*	25.75
Marker index	*MI*	*25.52*

AFLP banding patterns revealed a total number of 171 bands for all primer combinations, ranging in size from 120 to 1000 bp (Figs. 1 and 2). The results obtained will be discussed hereafter.

- Combination E-AGC/M-CAA

This combination exhibited a total of 35 bands ranging in size from 180 to1000 bp (Fig. 1), out of which 13 bands were monomorphic while the remainder were polymorphic bands with polymorphism percentage 63% (Table 4). Nine bands were negatively specific (absent) at band sizes (720, 670, 610, 540, 510, 440, 420, 380, and 265 bp), and the remaining polymorphic bands were positive specific. Six positive specific bands were unique for the cultivated *Thymus vulgaris* corresponding to band sizes (620, 570, 560, 500, 220, and 210 bp). Five positive bands were specific for *Thymus capitatus* (740, 320, 250, 240, and 180 bp) while two bands are unique for *Thymus decussates* (690 and 300 bp).

b. Combination E-AGC/M-CAC

**Table 4:** The AFLP primer combinations, selective nucleotides, number of total bands polymorphic bands, and fragment size range for *Thymus* species

Primer comb.	Selective nucleotides		Number of bands			Polymorphism percentage	Fragment size Range scored
	EcoRI	MseI	Total	Polymorphic	Monomorphic		
E-AGC/M-CAA	AGC	CAA	35	22	13	63%	180–1000 bp
E-AGC/M-CAC	AGC	CAC	23	8	15	35%	250–1000 bp
*E-AGG/M-CTC*	AGG	CTC	62	18	44	35%	120–830 bp
E-ACC/M-CAT	ACC	CAT	51	21	30	35%	120–790 bp
Total			171	69	102		
Average			42	17	25	42%	

The combination E-AGC/M-CAC produced the smallest number of bands (23 bands) relative to the other combinations; their sizes ranged from 250 to 1000 bp (Fig. 1). The number of monomorphic bands was 15, and only 8 bands were polymorphic ones showing polymorphism percentage 35% (Table 4). The positive specific bands were six bands, out of which four bands were specific for *Thymus decussates* (Benth.) species (sample C) at band sizes (390, 295, 270, and 250 bp) while band of size 520 bp was positively specific for sample (B) which represents *Thymus Capitatus* (L.). Band of size 490 bp was unique for sample (A) corresponding to the cultivated species *Thymus vulgaris*.

c. Combination E-AGG/M-CTC

It resulted in the largest number of bands (62) ranged between 120 and 830 bp (Fig. 2). The monomorphic bands were 44 bands, and the polymorphic ones were 18 bands (Table 4) with polymorphism percentage 35%. Seven bands were positive specific, three of which (nos. 19, 32, and 33) were unique for the cultivated species (*Thymus vulgaris*) with band sizes (385, 280, and 270), respectively. Two bands (550 and 160 bp) were specific for sample (B) representing the wild type (*Thymus capitatus* L.). The remaining 2 bands at (660 and 210 bp) were specific for sample (C) representing the wild type (*Thymus decussatus* Benth.). Eleven negative specific bands were detected, seven of them were for sample A (*Thymus vulgaris*) at 830, 630, 400, 285, 215, 155, and 130 bp. Three negative bands were for sample (C) at 700, 190, and180, and only one negative band at 150 bp is specific for sample (B).

d. Combination E-ACC/M-CAT

The combination E-ACC/M-CAT produced a total number of 51 bands ranging in size from 120 to 790 bp with polymorphism percentage 35% (Fig. 2). The monomorphic bands were 30 bands while the polymorphic ones were 21 bands (Table 4), of which 3 bands were unique for sample (C) (*Thymus decussatus* Benth.) at band sizes 790, 550, and 240 bp. Two bands were specific for sample (A), *Thymus vulgaris*, at band sizes 265 and 230 bp. Bands at 770, 400, 345, 270, 185, 175, 170, 153, and 150 bp were negatively specific for the studied *Thymus* species. Seven of them were negatively specific for sample (A) at 680, 630, 600, 385, 225, 200, and 150 bp. Five negative bands were specific for sample (C) at 345, 195, 175, 170, and153 bp while at 770, 400, and 185 bp negative specific bands were detected for sample (B).

The PIC values based on AFLP results of *Thymus* species were calculated. All results and the informativeness of the AFLP markers used for *Thymus* species genomes were depicted in Table 5. The PIC values were very high ranging between 0.96 and 0.99 with an average of 0.97.

**Table 5:** The PIC value and other information obtained from AFLP using four primer combinations for *Thymus* species

Parameters and their abbreviations		AFLP
Number of primer combinations	*U*	4
Number of non-polymorphic bands	*n*_*np*_	102
Number of polymorphic bands	*n*_*p*_	69
Average number of polymorphic bands/assay unit	*n*_*p*_*/U*	17
Number of loci	*L*	171
Number of loci/assay unit	*n*_*u*_	43
Total number of effective alleles	*N*_*e*_	*198*
Total banding pattern	*Bp*	27
Effective number of patterns/ assay unit	*P*	7
Min range of PIC value	*PIC*	0.96
Max range of PIC value	*PIC*	0.99
Average of PIC	*PIC*	*0.97*
Fraction of polymorphic loci	*β*	0.40
Assay efficiency index	*A*_*i*_	49.4
Effective multiplex ratio	*E*	17.25
Marker index	*MI*	*16.7*

### Data analyses

Applying the statistical package for social science program (SPSS) ver. 17 on the obtained results of the studied *Origanum* species and *Thymus* species using AFLP technique, the results were as follows:

- *Origanum* species

The similarity matrix of *Origanum* species (Table 6) revealed that the highest similarity percentage (86%) was between samples no. 4 and no. 5 representing *Origanum syriacum* L. *subsp. sinaicum* (Boiss.) collected from Saint Catherine region. However, the lowest similarity percentage (70%) was between samples no. 2 and no. 5.

**Table 6:** Genetic similarity matrix within *Origanum* samples based on AFLP data

Sample no.	*Origanum vulgare*	*Origanum Syriacum* L. *subsp. sinaicum* (Boiss.)			
	1	2	3	4	5
1					
2	83				
3	82	83			
4	71	72	1		
5	72	70	1	86	

**Fig. 3:**
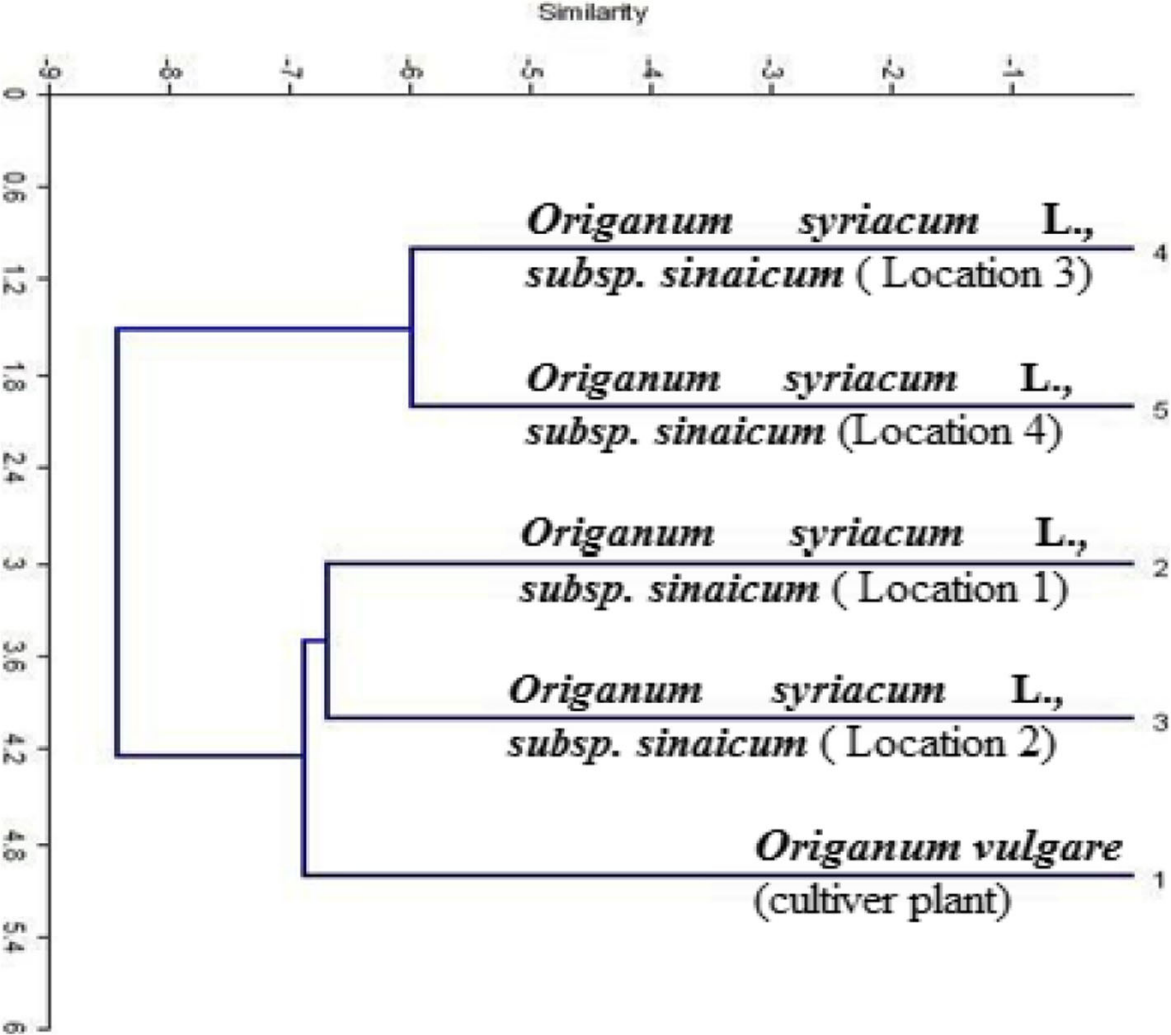
The genetic relatedness within (1) *Origanum vulgare* (cultivar plant collected from Kirdasa region) and (2–5) *Origanum syriacum* L. *subsp. sinaicum* (Boiss.) (wild type collected from different locations around Saint Catherine Monastery) based on AFLP results

The resulted dendrogram (Fig. 3) consisted of two main clusters. The first cluster branched into two groups, group 1 included sample no. 1 corresponding to the cultivated *Origanum* species (*Origanum vulgare*) while group 2 comprised samples no. 2 and 3 representing *Origanum syriacum* L*. subsp. sinaicum* (Boiss.) collected from different sites in Saint Catherine. However, the second cluster included samples no. 4 and no. 5 representing *Origanum syriacum* L. *subsp. sinaicum* (Boiss.) species collected from different sites in Saint Catherine.

b. *Thymus* species

The similarity matrix of *Thymus* species (Table 7) showed that the closest relationship was between sample B and sample (C) (86%). In contrast, the lowest similarity (81%) was between the cultivated type represented by sample (A) and sample (C).

**Table 7:** Genetic similarity matrix within the studied *Thymus* species based on AFLP data

Sample no.	A *T. vulgaris*	B *T. capitatus* L.	C *T. decassates* Benth.
A			
B	83		
C	81	86	

**Fig. 4:**
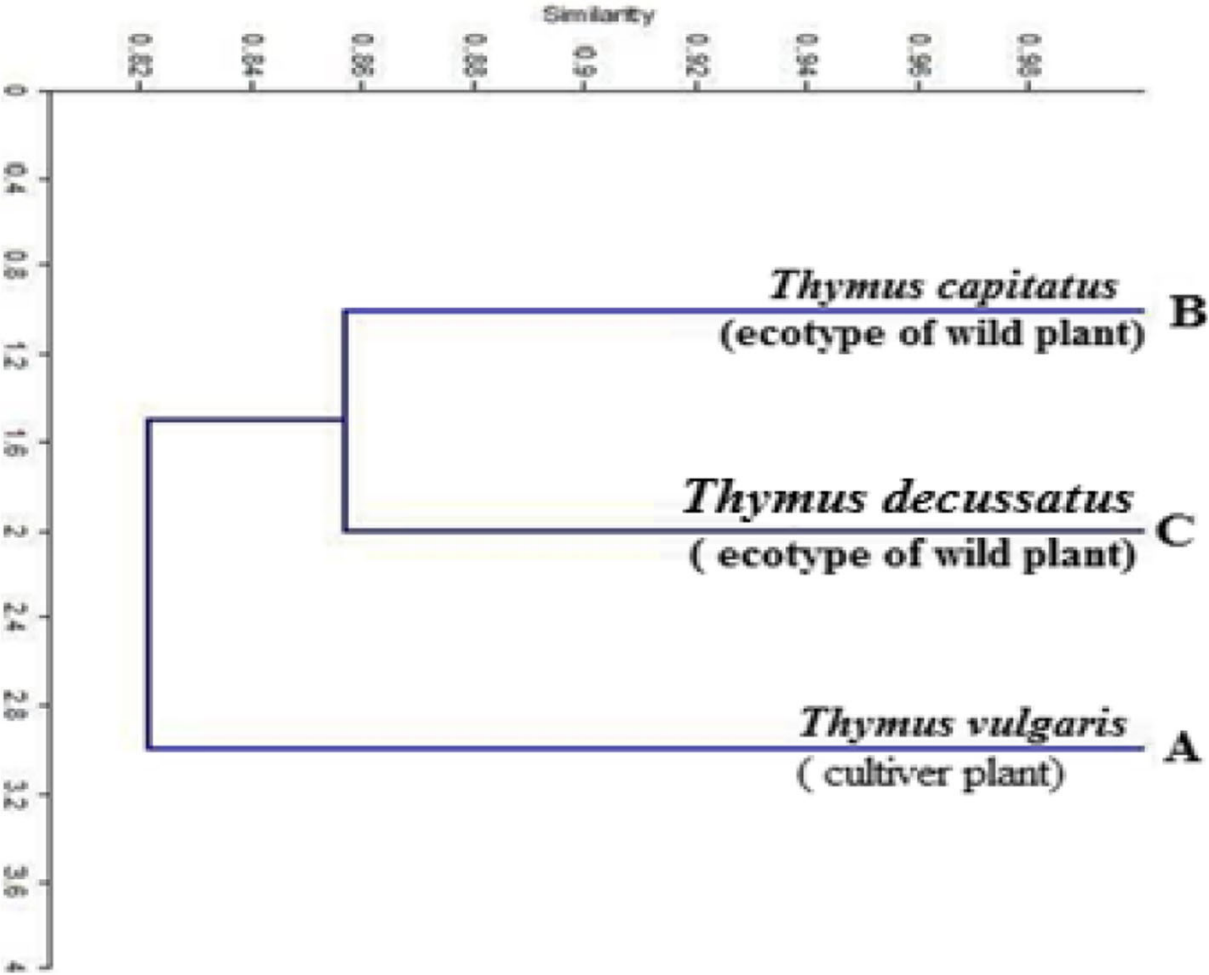
The genetic relatedness within (A) *Thymus vulgaris* (cultivated type collected from Kirdasa region), (B) *Thymus capitatus* (wild type collected from North Coast), and (C) *Thymus decassatus* (wild type collected from Saint Catherine) based on AFLP results

The obtained dendrogram (Fig. 4) divided the studied *Thymus* species into two main clusters. The first cluster included the cultivated type of *Thymus* (*Thymus vulgaris*), while the second cluster included the two wild species (*Thymus capitatus* L. and *Thymus decussates* Benth.).

## Discussion

The previous results of the studied *Origanum* species revealed that the monomorphic bands all over primer combinations are considered species-specific markers for *Origanum* species regardless of its type (cultivated or wild) or sampling location. Moreover, the primer combination *E-AGG/M-CTC* was more efficient to produce large numbers of amplified fragments (67 bands) with high levels of polymorphisms (46 polymorphic bands with 69% polymorphism percentage) compared to the other combinations. In contrast, the primer combination *E-AGC/M-CAC* was not useful in genetic diversity analysis of *Origanum* species (29 bands, 0% polymorphism). Overall primer combinations there were distinctive bands for *Origanum vulgare* (cultivated type) over *Origanum syriacum* (wild type) samples as mentioned previously. The results became more evident by the constructed dendrogram based on genetic distances where *Origanum syriacum* samples collected from different locations in Saint Catherine were more close to each other than *Origanum vulgare*.

**Fig. 5:**
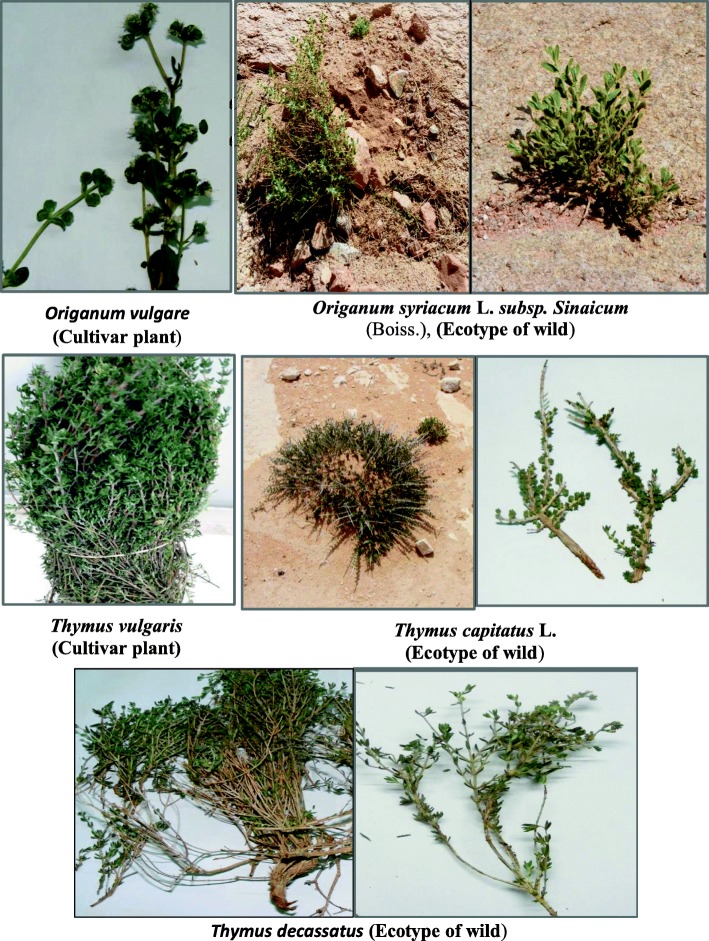
Showing the *Origanum* species and *Thymus* species under study

On the other hand, the obtained results of *Thymus* species under study showed that the monomorphic bands are characteristic for the studied species. In addition, the primer combination E-AGG/M-CTC was informative since it produced a large number of amplified fragments (62 bands). However, the primer combination E-AGC/M-CAA resulted in a less number of bands (35 bands) but the polymorphism percentage was moderately high relative to other primer combinations clarifying its usefulness and ability to detect polymorphisms within the studied species. Furthermore, the resulted dendrogram separated the cultivated type (*Thymus vulgaris*) from the two wild types (*Thymus capitatus* and *Thymus decussatus*). The studied *Origanum* and *Thymus* plant species are depicted in Fig. 5.

These findings were in agreement with [37] who studied the genetic diversity of *Ambrosia trifida* using AFLP markers, [12] who analyzed the genetic polymorphisms in *wild Nicotiana* species and *Cuban cultivated tobacco* using AFLP. Likewise, with Aparajita and Rout [3] who used AFLP to ascertain the extent of genetic diversity and relatedness in *Albizia* species. Other studies were conducted by [35] who investigated the genetic relationships among *Texas bluegrass* genotypes and [25]. Additionally [36] who used AFLP to study the molecular fingerprinting of the Egyptian medicinal plant *Cocculus pendulus* and [17] who constructed a DNA fingerprinting for some Egyptian date palm cultivars.

## Conclusion

The obtained results of *Origanum* and *Thymus* species may be rendered to the variations in morphology and chemical composition of the studied species. Noteworthy to mention that the genetic diversity in plants is not only related to internal genetic background and reproductive system but also affected by population structure, spatial distribution pattern, and reproduction mode [21].

Finally, the AFLP technique is reliable and useful technique to detect genetic variations and clarify the genetic relationships among the Egyptian *Thymus* and *Origanum* species. Moreover, the obtained results could be helpful for any improvement of cultivars and biodiversity maintenance and restoration of these genera.

## Data Availability

All plant material was identified and collected from their natural habitats by authors and authenticated by Dr. Yousri Abd-Elhady, Ecology and Range Management Department, Desert Research Center.

## Abbreviations

AFLP: Amplified fragment length polymorphism
DNA: Deoxyribonucleic acid
PIC: Polymorphism information content
SSR: Simple sequence repeat
SNPs: Single nucleotide polymorphisms
MseI: Restriction endonuclease enzyme isolated from *Micrococcus* species
EcoRI: Restriction endonuclease enzyme isolated from *Escherichia coli* species

## References

[1] Abdelkader MA, Mohamed NZ (2012). Evaluation of protective and antioxidant activity of thyme (*Thymus vulgaris*) extract on paracetamol-induced toxicity in rats. Australian J Basic Appl Sci.

[2] Al-Samarai FR, Al-Kazaz AA (2015). Molecular markers: an introduction and applications. Eur J Mol Biotechnol.

[3] Aparjita S, Ranjan RG (2010). Molecular analysis of Albizia species using AFLP markers for conservation strategies. J Genet.

[4] Assi, R (2007). MP Threat Analysis and Threat Reduction Assessment Report. Conservation and sustainable use of medicinal plants in arid and semi-arid ecosystems project.

[5] Ayesh BM, Abed AA, Faris DM (2014). vitro inhibition of human leukemia THP-1 cells by Origanum syriacum L. and *Thymus vulgaris* L. extracts. BioMed Central Res Notes.

[6] Begnini KR, Nedel F, Lund RG, Carvalho PH, Rodrigues MR, Beira FT (2014). Composition and antiproliferative effect of essential oil of *Origanum vulgare* against tumor cell lines. Journal of Medicinal Food..

[7] Bostancıoğlu RB, Kürkçüoğlu M, Başer KH, Koparal AT (2012). Assessment of anti-angiogenic and anti-tumoral potentials of *Origanum onites* L. essential oil. Food and Chemical Toxicology..

[8] Boulos L (2002). Flora of Egypt. Volume 3: Verbenaceae-Compositae.

[9] Chial H (2008) DNA fingerprinting using amplified fragment length polymorphisms (AFLP). Nat Educ 1(1)

[10] Chishti S, Kaloo ZA, Sultan P (2013). Medicinal importance of genus *Origanum*: A review. Journal of Pharmacognosy and Phytotherapy..

[11] Dhutmal RR, Mundhe AG, More AW (2018). Molecular markers techniques: a review. Int J Curr Microbiol App Sci Issue.

[12] Dominguez Y, Alvarez SP, Tapia MAM, Medina JAC, Ardisana EFH (2015) Analysis of genetic polymorphism in wild Nicotiana species and Cuban cultivated tobacco (solanaceae) through AFLP. Biotechnologia Aplicada 35(2)

[13] Ebrahimi M, Mokhtari A, Amirian R (2018). A highly efficient method for somatic embryogenesis of *Kelussia odorotissima* Mozaff., an endangered medicinal plant. Plant Cell. Tissue and Organ Culture.

[14] El Babili F, Bouajila J, Souchard JP, Bertrand C, Bellvert F, Fouraste I (2011). Oregano: chemical analysis and evaluation of its antimalarial, antioxidant, and cytotoxic activities. Journal of Food Science..

[15] El Gendy A, Leonardi M, Mugnaini L, Bertelloni F, Valentina VE, Nardoni S (2015). Chemical composition and antimicrobial activity of essential oil of wild and cultivated Origanum syriacum plants grown in Sinai. Egypt. Industrial Crops and Products..

[16] Elansary HO, Mahmoud EA (2015). Egyptian herbal tea infusions’ antioxidants and their antiproliferative and cytotoxic activities against cancer cells. Natural Product Research..

[17] El-Khishin DA, Adawy SS, EHA H, El-Itriby HA (2003). AFLP fingerprinting of some Egyptian date palm (*Phoenix dactylifera* L.) cultivars. Arab J Biotech.

[18] Ghosh S, Mandi SS (2018). Altitudinal effect in active principle content in Murraya koenigii (L) correlated with DNA fingerprinting study. Journal of Medicinal Plants Studies.

[19] Hansen M, Kraft T, Christiansson M, Nilsson N (1999). Evaluation of AFLP in Beta. Theor Appl Genet..

[20] Hashim S, Gamil M (1988). Plants and herbs between the Iraqi folk medicine and scientific research.

[21] Hu, Sh., Wu Sh, Wang Y, Zhao H and Zhang Y (2014). Genetic diversity and genetic structure of different types of natural populations in *Osmanthus fragrans* Lour. and the relationships with sex ratio, population structure, and geographic isolation. The Scientific World Journal, article ID 817080. 10.1155/2014/817080 PMC4243123 25436228

[22] Hussain Abdullah I., Anwar Farooq, Rasheed Shazia, Nigam Poonam S., Janneh Omar, Sarker Satyajit D. (2011). Composition, antioxidant and chemotherapeutic properties of the essential oils from two Origanum species growing in Pakistan. Revista Brasileira de Farmacognosia.

[23] Idrees, M and Irshad, M (2014). Molecular markers in plants for analysis of genetic diversity: a review. Eur Acad Res, Vol. (II); 1.

[24] Kokkini, F (1997). Taxonomy, diversity and distribution of *Origanum* species. In Proceedings of the IPGRI International Workshop on *Oregano*, Italy, Rome, pp:2-12.

[25] Koopman WJM, Zevenbergen MJ, Vander Berg RG (2001). Species relationship in Lactuceae s.l. (Lactuceae, Aseraceae) inferred from AFLP fingerprints Am. J. Bot..

[26] Kumar AS, Kavimani S, Jayaveera KN (2011). A review on medicinal plants with potential antidiabetic activity. International Journal of Phytopharmacology..

[27] Kumar P, Gupta VK, Misra AK, Modi DR, Pandey BK (2009). Potential of molecular markers in plant biotechnology. Plant Omics Journal.

[28] Leal Fernanda, Taghouti Meriem, Nunes Fernando, Silva Amélia, Coelho Ana Cláudia, Matos Manuela (2017). Thymus Plants: A Review—Micropropagation, Molecular and Antifungal Activity. Active Ingredients from Aromatic and Medicinal Plants.

[29] Liolios C, Graikou K, Skaltsa E, Chinou L (2010). Dittany of Crete: A botanical and ethnopharmacological review. Journal of ethnopharmacology..

[30] Liu J (2005). Oleanolic acid and ursolic acid: research perspectives. Journal of Ethnopharmacology..

[31] Marrelli M, Confortia F, Formisano C, Rigano D, Arnold NA, Menichini F (2015) Composition, antibacterial, antioxidant and antiproliferative activities of essential oils from three Origanum species growing wild in Lebanon and Greece. Natural Product Research.:1–5 10.1080/14786419.2015.1040993 26179294

[32] Moya-Hernández A, Bosquez-Molina E, Serrato-Díaz A, Blancas-Flores G, Francisco A (2018). Analysis of genetic diversity of *Cucurbita ficifolia* Bouché from different regions of Mexico, using AFLP markers and study of its hypoglycemic effect in mice. South African Journal of Botany.

[33] Omidbaigi R (2009). Production and processing of medicinal plants.

[34] Pirbalouti AG, Bistghani ZE, Malekpoor F (2015). An overview on genus Thymus. J Herbal Drugs.

[35] Renganayaki K, Read JC, Fritz AK (2001). Genetic diversity among Texas bluegrass genotypes (*Poa arachnifera* Torr.) revealed by AFLP and RAPD markers. Theor Appl Genet..

[36] Shadia AF, Fareida ME-S, Sengab AB, Naglaa MS, Osman AM, El-Shaimaa SE-D (2013). Molecular fingerprinting of the Egyptian medicinal plant *Cocculus pendulus*. Egypt J Med Hum Genet.

[37] Shao M, Fu D, Wang X, Liu Z, Qu B (2018). Genetic diversity of *Ambrosia trifida* L. as revealed by AFLP markers. Biotechnology Journal International.

[38] Smith JSC, Chin ECL, Shu H, Smith OS, Wall SJ (1997). An evaluation of the utility of SSR loci as molecular markers in maize (Zea mays L.): comparisons with data from RFLPs and pedigree. Theor Appl Genet..

[39] VERPOORTE R. (2000). Pharmacognosy in the New Millennium: Leadfinding and Biotechnology. Journal of Pharmacy and Pharmacology.

[40] Vos P, Hogers MB, Reijans MR, Vandelee T, Hornes M, Frijters A, Pot J, Peleman J, Kuiper M, Zabeau M (1995). AFLP: a new technique for DNA-fingerprinting. Nucl Acids Res..

[41] Vos P and Zabeau M (1993). Selective restriction fragment amplification: a general method for DNA fingerprinting. European Patent Office, publication 0 534 858 A1, bulletin 93/13.

[42] Vuylsteke M, Peleman JD, Eijk MJT (2007). AFLP technology for DNA fingerprinting. Nature Protocols.

[43] WCSP; World Checklist of Selected Plant Families (2017). http://wcsp.science.kew.org/home.do.

[44] Zhang X, Nick G, Kaijalainen S, Terefework Z, Paulin L, Tighe SW, Graham PH, Lindström K (1999). Phylogeny and diversity of Bradyrhizobium strains isolated from the root nodules of peanut (*Arachis hypogaea*) in Sichuan, China. Syst Appl Microbiol..

